# Predicting the Risk of Future Multiple Suicide Attempt among First-Time Suicide Attempters: Implications for Suicide Prevention Policy

**DOI:** 10.3390/healthcare10040667

**Published:** 2022-04-02

**Authors:** I-Li Lin, Jean Yu-Chen Tseng, Hui-Ting Tung, Ya-Han Hu, Zi-Hung You

**Affiliations:** 1 Department of Radiology, Ditmanson Medical Foundation Chia-Yi Christian Hospital, Chiayi 600, Taiwan; 05528@cych.org.tw; 2 Department of Public Affairs, Fo-Guang University, Yilan 262, Taiwan; yctseng@mail.fgu.edu.tw; 3 Department of Information Management, National Chung Cheng University, Chiayi County 621, Taiwan; sunshinetingo@gmail.com; 4 Department of Information Management, National Central University, Taoyuan City 320, Taiwan; yhhu@mgt.ncu.edu.tw; 5 Department of Nephrology, Chiayi Branch, Taichung Veterans General Hospital, Chiayi 600, Taiwan

**Keywords:** multiple suicide attempt, supervised learning, decision tree, artificial neural network

## Abstract

Suicide is listed in the top ten causes of death in Taiwan. Previous studies have pointed out that psychiatric patients having suicide attempts in their history are more likely to attempt suicide again than non-psychiatric patients. Therefore, how to predict the future multiple suicide attempts of psychiatric patients is an important issue of public health. Different from previous studies, we collect the psychiatric patients who have a suicide diagnosis in the National Health Insurance Research Database (NHIRD) as the study cohort. Study variables include psychiatric patients’ characteristics, medical behavior characteristics, physician characteristics, and hospital characteristics. Three machine learning techniques, including decision tree (DT), support vector machine (SVM), and artificial neural network (ANN), are used to develop models for predicting the risk of future multiple suicide attempts. The Adaboost technique is further used to improve prediction performance in model development. The experimental results show that Adaboost+DT performs the best in predicting the behavior of multiple suicide attempts among psychiatric patients. The findings of this study can help clinical staffs to early identify high-risk patients and improve the effectiveness of suicide prevention.

## 1. Introduction

The world has paid attention to a public health issue about suicide. According to an estimation from the World Health Organization, about 1 million people commit suicide in the world every year, and 60% of them occur in Asia. Suicide attempts or death by suicide can lead to huge losses in national productivity, the economy, and health care spending [1]. Therefore, countries around the world have adopted different suicide prevention strategies to reduce the incidence of suicide.

The causes of suicide are often complex, and no single factor can provide an adequate explanation. Joiner (2005) proposed that the composition of suicidal ideation must have the following two elements: thwarted belongingness and perceived burdensomeness [2]; suicidal ideation combined with the acquired capability for suicide constitutes a fatal suicide attempt. Different suicide ideation risk factors have been explored in past studies, such as mental illness, traumatic stress, substance use and impulsivity, chronic pain and illness, or social isolation.

Previous studies indicated that patients who had a past suicide attempt history could be treated as a high-risk group for multiple suicide attempts. Wong et al. (2008) pointed out that suicide attempters have a high probability of committing suicide again [3]. Chastang et al. (1997) and Scoliers et al. (2009) mentioned that up to 40% to 55% of suicide attempters who have had past suicide attempts history [4,5]. Lewinsohn (1994) pointed out that the risk period of multiple suicides occurs within three months after the first suicide attempt [6]. When suicidal behavior occurs, the probability of having multiple suicide attempts increases by 32%. Liaw and Chu (2009) indicated that a large portion of the cases of death by suicide have been diagnosed with psychiatric disorders [7].

In clinical practice, the primary methods of assessing suicide attempts among psychiatric patients were to use the general Brief Symptom Rating Scale (BSRS-5) or Pierce Suicide Intent Scale (PSIS) rating scales. Goto et al. (2010) further improved this rating scale to predict repeated suicide behavior in psychiatric patients [8]. However, Sjöström et al. (2009), Suokas et al. (2009), Gibb et al. (2009), and Belshaw et al. (2012) mentioned that these two rating scales do not focus on evaluating high-risk groups of multiple suicide attempts due to different psychiatric disorders [9,10,11,12].

Although previous studies have used various approaches to explore factors affecting psychiatric patients, suicides, or related risk factors, the analysis of multiple suicide attempts in psychiatric patients has not been explored. In addition, the sample datasets of previous studies are small due to the difficulty of obtaining patient data. Therefore, this study considers a large administrative database to establish a prediction model for predicting future multiple suicide attempts in psychiatric patients using data mining technologies. The psychiatric patient dataset was collected from the National Health Insurance Research Database (NHIRD) in Taiwan. Two data mining techniques, including decision tree (DT), support vector machine (SVM), and artificial neural network (ANN), were used to construct the prediction models and to find out the critical factors of future multiple suicide attempts. Developing a suicide attempt prediction model based on patients’ medical behavior history can help effectively identify future multiple-suicide attempt patients. If one can accurately identify these patients, the consumption of medical care resources can be largely decreased.

## 2. Materials and Methods

### 2.1. Data

The dataset was retrieved from the NHIRD, a nationwide representative claims database. As of 2020, the National Health Insurance (NHI) program covered more than 99% of the population of 23 million people in Taiwan. NHIRD contains complete information about clinical visits, including prescription details and diagnostic codes based on the International Classification of Disease, Ninth Revision, Clinical Modification (ICD-9-CM). The NHIRD is managed by Taiwan’s National Health Insurance Administration (NHIA). The investigated patient group was obtained from the Longitudinal Health Insurance Database 2010 (LHID 2010), which is a subset (i.e., claims data of one million randomly sampled enrollees) released by NHIA.

The study group consisted of suicide attempters with at least one psychiatric visit (i.e., psychiatric patients having ICD-9-CM Diagnosis Code: E950~E959). Because our purpose is to identify future multiple suicide attempters from those psychiatric patients with a history of suicide attempts, data records with any of the following conditions were excluded: (1) patients without psychiatric visit records, and (2) patients died after first suicide attempt. After data filtering by the two conditions, a total of 523 psychiatric patients were obtained. By counting the number of suicide attempts, the patients can be partitioned into two groups, including 238 multiple suicide attempters and 285 non-multiple suicide attempters.

### 2.2. Variables

Our study classified the risk factors of suicide (i.e., independent variables) into six categories, including patients’ psychiatric disorder history, personal characteristics, medical history, physician characteristics, and hospital characteristics. Patients’ psychiatric disorder history variables record the occurrence of various mental illnesses in psychiatric patients before their first suicide attempt. The basic patient characteristics include general demographic variables [1]. Personal medical history includes a history of non-psychiatric illness before medical treatment [13,14]. The physician characteristics include the results of the psychic disorders scale plus a history of psychic disorders [15,16]. Suicide history includes suicide-related variables such as the method and place of first suicide attempt. The characteristics of medical behavior include environmental variables, pharmacological variables, and other medical behavior variables during medical treatment. The characteristics of physicians and institutions are related to the characteristics of the patient. The details are shown in Table 1.

### 2.3. The Investigated Machine Learning (ML) Techniques

This study considered both DT and the ANN techniques to develop the classification models. To further improve classification performance, the adaptive boosting (i.e., Adaboost) technique is also used in model development.

DT, also known as classification tree, is a classification algorithm with a tree structure [17,18,19]. A DT includes a root node and a number of internal nodes and leaf nodes. Each internal node represents an attribute, and each branch of an internal node represents an attribute value. The class label is given in each leaf node. The generation process of a DT consists of the following two phases: tree growing and pruning. At the phase of tree growth, a divide-and-conquer approach is used to select a suitable attribute as an internal-node of a tree, which partitions the training dataset into subsets. This process is recursively applied to each internal node until any of the stopping criteria is satisfied. In the end of tree growing, a class label is assigned to a leaf-node based on a majority vote. In the phase of tree pruning, DT uses a pre-pruning approach to reduce the tree size and thus avoid the over-fitting problem.

Gupta et al. (2011) and Huda et al. (2016) mentioned that ANN is one of the most well-known and representative supervised learning techniques [20,21]. Inspired by the biological neural systems, ANN consists of an input and an output layer with a number of hidden layers. Artificial neurons between two adjacent layers are connected; the neurons receive the inputs from the outputs of the front adjacent neurons and convert them into an output value through the transfer functions. At the end of the entire ANN structure, the output can be estimated in the output layer.

One of the most popular ANN methods is the multilayer feedforward neural network trained with the backpropagation algorithm [22,23]. In a multilayer feedforward neural network, neurons are directly connected in the input layer, hidden layer, and output layer, and there is no connection between any two neurons in the same layer. The key component in the ANN learning procedure is the connection weights between two neurons. The input and output layers are used to represent the input variables and output values, respectively. In training phase, the algorithm iteratively tunes the connection weights between neurons to minimize the difference between the estimated output value and the actual output. The learning process repeatedly executes the feedforward and backward phases until any stop criteria is reached.

Developed by Vapnik and the research team at the AT&T Laboratory, SVM is a well-known supervised learning method [24]. Given a training set, SVM first projects training instances into a high-dimensional vector space. Then, it attempts to find a hyperplane to separate the training data into two classes using structural risk minimization. Therefore, this hyperplane can be used to map a new test instance into one of the two subspaces and determine its class label.

In addition to the use of DT and ANN, this study further considered classifier ensembles to improve the prediction performance of the two classification techniques. Many of previous studies indicated that single classifier cannot always make a good prediction performance in noise datasets [23,25]. Freund and Schapire (1996) and Liu et al. (2014) mentioned that AdaBoost, one of the most famous meta-learners, iteratively applies a selected classification algorithm to generate multiple weak learners from the training dataset [18,26]. In each iteration, the weights of samples incorrectly classified by the current learner are increased for the next round of learning. In other words, AdaBoost encourages the selected classification algorithm to learn from the instances misclassified by the earlier iteration. After a sequence of classifiers is built, the final output is determined by combining the weighted sum of the generated classifiers. Previous studies have indicated that many classification algorithms combined with AdaBoost can achieve higher classification accuracy than the base classifiers.

### 2.4. Experimental Setup and Performance Measure

This study used Waikato Environment for Knowledge Analysis (WEKA) version 3.6.6 data mining software [27]. We performed DT (J48 module in WEKA), SVM (SMO module in WEKA), and ANN (MultilayerPerceptron module in WEKA) techniques to compare the accuracy of the prediction models. To optimize the prediction performance of the models, we further performed parameter selection by cross-validation (i.e., CVParameterSelection in WEKA) technique to search the best parameter settings. The tests of parameter optimization are listed in Table 2.

In all experiments, the 10-fold cross-validation was used to avoid overfitting. Specifically, the prediction models were trained using 9 of the 10 folds as training data and tested using the holdout testing data. To further explore the effects of the independent variables on the dependent variable, we used a feature selection module, named GainRatioAttributeEval, in WEKA to identify important independent variables. In the experimental evaluation of feature selection, we selected top 30 most important independent variables.

**Figure 1:**
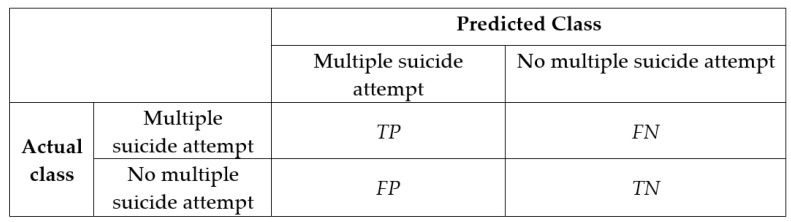
Confusion matrix.

To evaluate the efficacy of these prediction models, the accuracy, sensitivity, and specificity were evaluated using a confusion matrix (Figure 1). True positives (TP) indicate the number of patients correctly classified as having multiple suicide attempts, whereas true negatives (TN) mean the number of patients correctly classified as not having multiple suicide attempts. False positives (FP) indicate the number of patients incorrectly classified as having multiple suicide attempts, while false negatives (FN) mean the number of patients incorrectly classified as not having multiple suicide attempts. These metrics were determined using the following formulas: sensitivity = TP/(TP + FN), specificity = TN/(TN + FP), accuracy = TP + TN/(TP + FP + FN + TN).

## 3. Results

The descriptive statistics of the variables used in this study are shown in Table S1. There were 238 cases drawn from the group with multiple suicide attempts and 285 cases without multiple suicide attempts, resulting in 523 valid clinical cases in total. Among them, there were 202 male (38.6%) and 321 female (61.4%) patients.

Table 3 summarizes the prediction performance of each classifier. In the original dataset, the Adaboost+DT had the best prediction performance, which indicated a 0.971 overall prediction accuracy and a 0.965 specificity for the correct prediction of no multiple suicide attempts. However, Adaboost+SVM holds the best sensitivity (0.987) for the correct prediction of multiple suicide attempts. The second best is the ANN model, demonstrating a 0.979 sensitivity and a 0.933 specificity. The overall ANN model had a prediction accuracy of 0.954. We also noticed that, except for ANN, the classifiers supplemented with Adaboost can improve the prediction performance.

In the dataset with feature selection, the performance of Adaboost+DT was improved, which indicated a 0.987 sensitivity for the correct prediction of multiple suicide attempts and a 0.979 specificity for the correct prediction of no multiple suicide attempts. The overall Adaboost+DT model had a prediction accuracy of 0.983. The second best is the Adaboost+SVM model, where the accuracy, sensitivity, and specificity are 0.962, 0.987, and 0.940, respectively. The results from the dataset with feature selection also indicated that the prediction performance can be further improved by incorporating the Adaboost technique compared to those from the original dataset.

In addition, including redundant features in model training can lead to overfitting and reduced efficiency. Feature selection techniques narrow down the set of features to those most correlated with the target variable, resulting in simpler and more effective prediction models. The experimental results also indicated that, except for SVM, the accuracy of all classifiers improved after feature selection.

Based on the results shown in Table 3, the DT combined with the Adaboost technique is suggested as the best classifier for the prediction of multiple suicide attempts in psychiatric patients. The top five most important decision rules generated by Adaboost+DT are summarized in Table S2. The decision rules induced by the Adaboost+DT can be used for developing a decision support system of evaluating multiple suicide risk.

Even though the developed Adaboost+DT classifier can be used as a clinical decision support tool, identifying the most critical features associated with multiple suicide attempts is actually the key to quality prediction. Several features are found to be crucial in multiple suicide attempts. The importance of the investigated independent variables (i.e., the top 30) calculated by the GainRationAttributeEval module is shown in Table 4. The results indicated that physician experience with suicidal patients can be found to be the most important variable. If a suicide attempter visits an experienced physician, it will decrease the possibility of his/her future multiple suicide attempts.

## 4. Discussion

This study further discussed the important features listed in Table 4. We noticed that of the top 10 important variables, five of them belong to the category of patients’ psychiatric disorder history. This result is consistent with the studies of Chang et al. (2009), Juurlink (2004), and Malloy-Diniz et al. (2009) [28,29,30]. Suominen et al. (2009) found that severe depression may increase the risk of multiple suicide attempts [31]. Walrath et al. (2001) analyzed the clinical characteristics of four different suicide attempts among children with emotional disturbances in the National Institute of Mental Health-initiated Child and Adolescent Service System Program (CASSP). It includes the clinical features of no recent attempted suicide, one-time suicide attempts, multiple suicide attempts, and no suicide attempts [32]. Lizardi et al. (2009) predicted multiple suicide with or without a family history of suicidal behavior [33]. The results of regression analysis indicated that having a family history of suicidal behavior predicts more suicide attempts. Lopez Castroman et al. (2011) found that affective disorders and anxiety disorders significantly increase the risk of frequent suicide attempts [34]. The risk of frequent suicide attempts was highest among middle-aged subjects and gradually decreased with increasing age at the onset of the first attempt. Vajda and Steinbeck (2000) indicated that adolescents with either sexual abuse, non-affective psychotic disorders, or drug abuse have a higher risk of multiple suicide attempts within 12 months [35].

Studies have shown that depressed patients with a history of suicidal behavior have more suicide attempts [3,13,14]. Gibb et al. (2009) compared the depression characteristics of psychiatric outpatients with multiple suicide attempts [11]. The results indicated that multiple suicide attempters have higher levels of depressive symptoms at psychiatric clinical diagnosis. It also showed that multiple suicide attempters were more likely to have a history of substance abuse, depression, and domestic violence. Melhem et al. (2019) developed a prediction model for suicide attempts using depression symptoms [14]. A number of critical predictors were found, such as a history of unipolar and bipolar disorders.

We also have the same findings as past research that sleep disorders are also a significant indicator of suicide attempts [36,37]. Sjöström et al. (2007) showed that two-thirds of multiple suicide attempters have nightmare disease; patients with a consistent history of sleep disorders may be an indicator of multiple suicide attempters [38]. Sjöström et al. (2009) indicated that persistent sleep disorders increase the risk of multiple suicide attempts [9]. Chellappa and Araújo (2007) and Bishop et al. (2013) have pointed out that sleep disturbance is related to suicidal behavior [39,40]. Wang et al. (2019) conducted a systematic review and meta-analysis to explore the association between sleep disorders and suicide attempts [37]. They found sleep disorders, particularly nightmares and insomnia, increase the risk of suicide attempts. Dolsen et al. (2021) investigated the associations between sleep duration, insomnia, and inflammation on suicide attempt [41]. A large sample of adults with depression or anxiety was included. The result showed that the group with a short sleep duration had a higher probability of suicide attempt compared to the group with a normal sleep duration. Geoffroy et al. (2021) performed structural equation modeling using national epidemiological survey data to examine the effects of three sleep complaints on the 3-year occurrence of attempting suicide [42]. They concluded that sleep complaints increase the risk of suicide attempts and is suggested to be included in suicide risk assessments.

Explainable artificial intelligence (XAI) has received much attention in healthcare in recent years [43,44,45]. Generally, the XAI methods follow the following three principles: transparency, interpretability, and explainability [46]. The transparency of a ML algorithm is high if the design of the proposed method can be clearly depicted. The interpretability considers the connections between the data and the ML models; the goal is to understand how the ML models make predictions based on the collected training data. The explainability further involves human experts to validate decisions, improve ML models, and confirm newly discovered knowledge.

DT has been recognized as a white-box ML algorithm with high transparency and interpretability. Compared with other ML algorithms, DT is efficient, intuitive, and easy to apply in many real-life applications. Although conventional DT approaches have been proven to be prone to overfitting, this problem can be greatly mitigated by incorporating classifier ensemble techniques.

The developed Adaboost+DT, a decision tree-based classifier ensemble, has been validated as an effective model for classifying multiple suicide attempts. It also satisfies all the basic requirements of XAI. As shown in Table S2, the knowledge extracted by the Adaboost+DT is in the form of IF-THEN rules. Hospitals can easily build problem-oriented expert systems by incorporating these rules into an expert system shell. When an outpatient goes to the hospital for treatment, the physician can query the cloud medical records using the patient’s health insurance IC card and automatically import the data into the expert system to generate multiple suicide risk assessment results. Therefore, the decision rules generated from our Adaboost+DT model can be of assistance to clinical practitioners in making better medication decisions for psychiatric patients.

## 5. Conclusions

This study adopted a retrospective research method and used a representative health administration database to construct multiple suicide attempt prediction models. We considered a large number of features extracted from the database, including aspects of psychiatric disorder history, personal information, medical history, medical behavior, physician, and hospital. Two supervised learning techniques were selected to develop the prediction models. The results indicated that Adaboost+DT performs the best in predicting the behavior of multiple suicide attempts among psychiatric patients. Several critical factors were also uncovered.

Suicide prevention is an important topic in public health and mental health. Psychiatric patients with a suicide history are among the most concerned groups. Using patients’ historical medical records, our proposed prediction model can help to identify high-risk groups for multiple suicide attempts in advance. The results suggested that clinical staff should be aware of middle-aged patients with major depressive disorder, alcohol addiction syndrome, or specific nonorganic insomnia. In addition, high-risk factors for multiple suicidal behaviors included the length of hospital stay before suicide and the degree of urbanization of the hospital location.

Our findings can provide recommendations to the suicide prevention authorities. For example, the method proposed in this study can construct different models for patients of different ages, seasons, medical histories, and even different regions to assess their risk of multiple suicide behaviors. We can, therefore, define different suicide prevention strategies for each group and reduce the occurrence of multiple suicide behaviors.

Finally, it is worth noting that our study is not without limitations. First, some important features are not available in NHIRD. For example, assessment scales for various psychiatric disorders and patient complaints may contain signals of potential suicidal behavior. Incorporating complete patient assessment data will help optimize the prediction models. Second, there are still more important features that can be extracted from other data sources, such as electronic health records or self-harm monitoring databases. These databases can be linked to include more features and construct more effective prediction models.

## Figures and Tables

**Table 1:** Research variable.

Category	Variable
Psychiatric disorder history	Organic psychosis, Schizophrenia, Schizoaffective disorders, Major depressive disorder, Bipolar disorder, Other emotional psychosis, Delusional state, Other nonorganic psychoses, Psychosis with origin specific to childhood, Depression, anxiety, Other neurotic disorder, Personality disorder, Sexually biased disorder, Alcohol addiction syndrome, Drug addiction, Drug abuse, Alcohol abuse, Psychogenic physiological dysfunction, Specific nonorganic insomnias, Other specific symptoms or Symptoms, Acute psychological stress response, Environmental adaptation disorders, Specific non-psychiatric disorders after organic brain injury, Behavioral disorders, Mood disorders in children and adolescents, Hyperkinetic reaction of childhood, Specific developmental delay, Psychiatric factors related to other specific diseases, Mental retardation.
Personal characteristics	Gender, Age, Place of residence, Insured grade group, Employment status.
Medical history	Lung cancer, Stomach cancer, Oral cancer, Breast cancer, Blood cancer, History of domestic violence, Major injuries, Number of psychiatric disorders diagnoses.
Medical behavior	Season, Month, Number of visits in psychiatric clinic, Day of hospital stays, The use of antipsychotic drugs, Number of comorbidities, The voluntary discharge of patients.
Physician	Physician gender, Physician age, Physician working experience, Physician experience with suicide patients.
Hospital	Hospital level, Hospital ownership, Teaching hospital, Hospital location.

**Table 2:** Parameter settings.

Technique	Parameters	Test Range	Increment
DT	Minimum number of instances per leaf Confidence factor	2–20 0.1–0.3	1 0.05
ANN	Learning rate Momentum factor Maximum number of epochs	0.3–0.5 0.1–0.5 300–700	0.05 0.05 100
SVM	Kernel	Polykernel, RBF Kernel	

**Table 3:** Prediction performance of different classifiers.

Dataset	Metric	DT	SVM	ANN	Adaboost+ DT	Adaboost+ SVM	Adaboost+ ANN
Original dataset (60 variables)	Sensitivity	0.912	0.924	0.979	0.979	0.992	0.979
	Specificity	0.923	0.884	0.933	0.965	0.912	0.933
	Accuracy	0.918	0.902	0.954	0.971	0.948	0.954
Dataset with feature selection (30 variables)	Sensitivity	0.941	0.866	0.983	0.987	0.987	0.983
	Specificity	0.895	0.888	0.940	0.979	0.940	0.937
	Accuracy	0.916	0.878	0.960	0.983	0.962	0.958

**Table 4:** The importance of investigated independent variables.

Category	Attribute	Values	Rank
Psychiatric disorder history	Organic psychosis	0.049642	16
	Schizophrenia	0.112739	5
	Schizoaffective disorders	0.099837	7
	Major depressive disorder	0.077159	10
	Delusional state	0.065465	13
	Depression	0.016177	23
	Anxiety	0.008136	28
	Personality disorder	0.039829	18
	Neurotic disorder	0.023141	22
	Alcohol addiction syndrome	0.036201	19
	Drug addiction	0.064032	14
	Specific non-organic sleep disorders	0.062536	15
	Other specific symptoms or symptoms	0.069882	12
	Specific non-psychiatric disorders after organic brain injury	0.115462	3
	Psychiatric factors related to other specific diseases	0.115462	4
Personal information	Age	0.010442	25
	Place of residence	0.010189	26
	Insured grade group	0.035000	20
Medical history	Major injuries	0.082401	9
	Number of psychiatric disorders’ diagnoses	0.047797	17
Medical behavior	Department	0.004705	29
	Month	0.009877	27
	Count of psychiatric treatment	0.196394	2
	Day of hospital stays	0.071561	11
	The use of antipsychotic drugs	0.110739	6
	Number of joint diseases	0.091155	8
Physician	Physician’s experience of diagnosis with suicide	0.245547	1
Hospital	Hospital grade	0.013388	24
	Hospital ownership	0.000626	30
	Hospital location	0.026316	21

## Data Availability

The datasets used in the current study can be accessed from the Taiwan National Health Insurance Research Database repository via application (accessed on 14 December 2020).

## Supplementary Materials

The following are available online at https://www.mdpi.com/article/10.3390/healthcare10040667/s1, Table S1: Descriptive Statistics, Table S2: Decision rules generated by Adaboost+DT.
