# Supplementary material for: Predicting the Risk of Future Multiple Suicide Attempt among First-Time Suicide Attempters: Implications for Suicide Prevention Policy
Source: Healthcare (Basel). 2022 Apr 2;10(4):667. doi: 10.3390/healthcare10040667 (PMC9032869; doi:10.3390/healthcare10040667)
Supplement: Supplementary file 1 [file healthcare-10-00667-s001.zip › healthcare-1606226-supplementary.pdf]

Supplemental Table

Table S1. Descriptive Statistics.

Category	Variable	Definition	Summary Statistics
**Psychiatric disorder history**	Organic psychosis	Y/N	Y: 87 (16.6%)<br>N: 436 (93.4%)
	Schizophrenia	Y/N	Y: 33 (6.3%)<br>N: 490(93.7%)
	Schizoaffective disorders	Y/N	Y: 24(4.6%)<br>N: 499(95.4%)
	Major depressive disorder	Y/N	Y: 217(41.5%)<br>N: 306(58.5%)
	Bipolar disorder	Y/N	Y: 0(0%)<br>N: 523(100%)
	Other emotional psychosis	Y/N	Y: 45(8.6%)<br>N: 478(91.4%)
	Delusional state	Y/N	Y: 25(4.8%)<br>N: 498(95.2%)
	Other nonorganic psychoses	Y/N	Y: 42(8%)<br>N: 481(92%)
	Psychosis with origin specific to childhood	Y/N	Y: 1(0.2%)<br>N: 522(99.8%)
	Depression	Y/N	Y: 276(52.8%)<br>N: 247(47.2%)
	Anxiety	Y/N	Y: 185(35.4%)<br>N: 338(64.6%)
	Other neurotic disorder	Y/N	Y: 316(60.4%)<br>N: 207(39.6%)
	Personality disorder	Y/N	Y: 50(9.6%)<br>N: 473(90.4%)
	Sexually biased disorder	Y/N	Y: 1(0.2%)<br>N: 522(99.8%)
	Alcohol addiction syndrome	Y/N	Y: 48(9.2%)<br>N: 475(90.8%)
	Drug addiction	Y/N	Y: 36(6.9%)<br>N: 487(93.1%)
	Drug abuse	Y/N	Y: 66(12.6%)<br>N: 457(87.4%)
	Alcohol abuse	Y/N	Y: 24(4.6%)<br>N: 499(95.4%)
	Psychogenic physiological dysfunctio	Y/N	Y: 93(17.8%)<br>N: 430(82.2%)
	Specific nonorganic insomnias	Y/N	Y: 375(71.7%)<br>N: 148(28.3%)
	Other specific	Y/N	Y: 52(9.9%)

Category	Variable	Definition	Summary Statistics
	symptoms or Symptoms		N: 471(90.1%)
	Acute psychological stress response	Y/N	Y: 25(4.8%)<br>N: 498(95.2%)
	Environmental adaptation disorders	Y/N	Y: 60(11.5%)<br>N: 463(88.5%)
	Specific non-psychiatric disorders after organic brain injury	Y/N	Y: 7(1.3%)<br>N: 516(98.7%)
	Behavioral disorders	Y/N	Y: 2(0.4%)<br>N: 521(99.6%)
	Mood disorders in children and adolescent	Y/N	Y: 0(0%)<br>N: 523(100%)
	Hyperkinetic reaction of childhood	Y/N	Y: 0(0%)<br>N: 523(100%)
	Specific developmental delay	Y/N	Y: 0(0%)<br>N: 523(100%)
	Psychiatric factors related to other specific diseases	Y/N	Y: 7(1.3%)<br>N: 516(98.7%)
	Mental retardation	Y/N	Y: 3(0.6%)<br>N: 520(99.4%)
**Personal information**	Gender	Male/Female	Male: 202(38.6%)<br>Female: 321(61.4%)
	Age	1: Childhood (0~14 yrs)<br>2: Teenage (15~24 yrs)<br>3: Adolescent (25~44 yrs)<br>4: MiddleAge (45~64 yrs)<br>5: Old (65+ yrs)	1: 3(0.6%)<br>2: 315(60.2%)<br>3: 123(23.5%)<br>4: 58(11.1%)<br>5: 24(4.6%)
	Place of residence	1: High degree of urbanization<br>2: Medium degree of urbanization<br>3: New town<br>4: General townships<br>5: Aging town<br>6: Agricultural tow<br>7: Remote towns<br>8: Others	1: 134(25.6%)<br>2: 127(24.3%)<br>3: 77(14.7%)<br>4: 69(13.2%)<br>5: 12(2.3%)<br>6: 11(2.1%)<br>7: 10(1.9%)<br>8: 83(15.9%)
	Insured grade group	1: < 17280(NTD)<br>2: 17281-22800(NTD)<br>3: 22801-28800(NTD)<br>4: 28801-36300(NTD)<br>5: 36301-45800(NTD)	1: 355(67.9%)<br>2: 128(24.5%)<br>3: 27(5.2%)<br>4: 6(1.1%)<br>5: 4(0.8%)

Category	Variable	Definition	Summary Statistics
		6: 45801-57800(NTD)	6: 3(0.6%)
		7: > 57800(NTD)	7: 0(0%)
	Employment status	Y/N	N: 438(83.7%)<br>Y: 85(16.3%)
**Medical history**	Lung cancer	Y/N	N: 523(100%)<br>Y: 0(0%)
	Stomach cancer	Y/N	N: 523(100%)<br>Y: 0(0%)
	Oral cancer	Y/N	N: 523(100%)<br>Y: 0(0%)
	Breast cancer	Y/N	N: 523(100%)<br>Y: 0(0%)
	Blood cancer	Y/N	N: 523(100%)<br>Y: 0(0%)
	History of domestic violence	Y/N	N: 523(100%)<br>Y: 0(0%)
	Major injuries	Y/N	N: 440(84.1%)<br>Y: 83(15.9%)
	Number of psychiatric disorders diagnose	0~13	μ=4.02, σ=2.637
**Medical behavior**	Department	1: General	1: 117(22.4%)
		2: Nephrology	2: 120(22.9%)
		3: Sugery	3: 46(8.8%)
		4: Orthopedics	4: 50(9.6%)
		5: Gastroenterology	5: 34(6.5%)
		6: Chest Medicine	6: 54(10.3%)
		7: Psychiatry	7: 15(2.9%)
		8: Other	8: 87(16.6%)
	Season	0: Spring	0: 141(27%)
		1: Summer	1: 146 (27.9%)
		2: Fall	2: 136 (26%)
		3: Winter	3: 100(19.1%)
	Month	1: January	1: 33(6.3%)
		2: February	2: 28(5.4%)
		3: March	3: 36(6.9%)
		4: April	4: 57(10.9%)
		5: May	5: 48(9.2%)
		6: June	6: 39 (7.5%)
		7: July	7: 43(8.2%)
		8: August	8: 64 (12.2%)
		9: September	9: 43(8.2%)
		10: October	10: 52(9.9%)
		11: November	11: 41(7.8%)
		12: December	12: 39(7.5%)
	Number of visits in		μ=1.15, σ=2.614

Category	Variable	Definition	Summary Statistics
	psychiatric clinic		
	Number of days in hospital stay		μ=6.84, σ=11.189
	The use of antipsychotic drugs	Y/N	N: 271(51.8%)<br>Y: 252(48.2%)
	Number of comorbidities		μ=4.17, σ=2.743
	Voluntary discharge of patients	Y/N	N: 451(86.2%)<br>Y: 72(13.8%)
**Physician**	Physician gender	Male/Female	Male: 473(90.4%)<br>Female: 50(9.6%)
	Physician age	1: Adolescent (25~44 yrs)<br>2: MiddleAge (45~64 yrs)<br>3: Old(65+ yrs)	1: 362(69.2%)<br>2: 159(30.4%)<br>3: 2(0.4%)
	Physician working experience	0~23	μ=5.13, σ=4.722
	Physician experience with suicide patients	0~8	μ=1.2, σ=2.168
**Hospital**	Hospital level	1: Medical center<br>2: Metropolitan hospital<br>3: Local hospital<br>4: Other	1: 154(29.4%)<br>2: 260(49.7%)<br>3: 109(20.8%)<br>4: 0(0%)
	Hospital ownership	1: Public<br>2: Private(non-profit)<br>3: Private(profit)<br>4: University hospital	1: 440(84.1%)<br>2: 83(15.9%)<br>3: 0(0%)<br>4: 0(0%)
	Teaching hospital	Y/N	Y: 403(77.1%)<br>N: 120(22.9%)
	Hospital location	1: High degree of urbanization<br>2: Medium degree of urbanization<br>3: New town<br>4: General townships<br>5: Aging town<br>6: Agricultural tow<br>7: Remote towns<br>8: Others	1: 152(29.1%)<br>2: 236 (45.1%)<br>3: 47(9%)<br>4: 75(14.3%)<br>5: 2(0.4%)<br>6: 0(0%)<br>7: 10(1.9%)<br>8: 1(0.2%)
**Dependent Variable**	Multiple suicide attempt	Y/N	Y: 238(45.5%)<br>N: 285(54.5%)

Table S2. Decision rules generated by Adaboost+DT.

Rule ID	Descriptions
1	If the psychiatric patients without specific nonorganic insomnias have been treated by psychiatrists without having suicide diagnosis experience, the number of hospital stays in the psychiatric department is less than 12 days, and the hospital location is in the township, then the patients have a high-risk of multiple suicide attempt.
2	If the psychiatric patients with specific nonorganic insomnias have been treated by psychiatrists without having suicide diagnosis experience, seek medical treatment in May, June, July or December, and the insurance coverage is less than NTD 17,280, then the patients have a high-risk of multiple suicide attempt.
3	If the psychiatric patients with specific nonorganic insomnias or organic psychopathic disorders or severe depression have been treated by psychiatrists without having suicide diagnosis experience, and the insurance coverage is less than NTD 17,280, then the patients have a high-risk of multiple suicide attempt.
4	If the psychiatric patients with specific nonorganic insomnias have been treated by psychiatrists without having suicide diagnosis experience, their insurance coverage is between NTD 17,281 and NTD 22,800, and the hospital location is in urban area, then the patients have a high-risk of multiple suicide attempt.
5	If the psychiatric patients with specific nonorganic insomnias have been treated by psychiatrists without having suicide diagnosis experience, their insurance coverage is between NTD 45,801 and NTD 57,800, then the patients have a high-risk of multiple suicide attempt.
